# Delayed cardiac tamponade following catheter ablation of frequent premature ventricular complexes: a case report

**DOI:** 10.1186/s12872-020-01642-7

**Published:** 2020-08-05

**Authors:** Xiaoyong Xu, Xianghong Meng, Fusheng Ma

**Affiliations:** 1 Department of Cardiovascular Disease, Ningbo Medical Treatment Centre Li Huili Hospital, No.57 Xingning Road, Ningbo, 315040 Zhejiang China; 2 Department of Medical Technology, Ningbo College of Health Sciences, Ningbo, Zhejiang China

**Keywords:** Cardiogenic shock, Delayed cardiac tamponade, Catheter ablation, Ventricular arrhythmia, Premature ventricular complex

## Abstract

**Background:**

Cardiac tamponade is a potentially fatal complication after catheter ablation of ventricular arrhythmias. It often happens during or shortly after the procedure and needs urgent treatment. Here, we present a very incredible case about delayed cardiac tamponade after ablation of premature ventricular complexes.

**Case presentation:**

A 66-year-old woman who underwent successful catheter ablation of right ventricular outflow tract origin premature ventricular complexes. Nineteen days after ablation, the patient experienced sudden syncope. Upon arriving at our hospital, she was “confused and shock”. Transthoracic echocardiography revealed hemorrhagic cardiac tamponade, which was considered due to a delayed tiny perforation in the heart induced by the previous ablation. Following an emergent pericardiocentesis to drain a 200 mL hemorrhagic effusion, the patient’s hemodynamics improved significantly. The patient was discharged after a 2-week hospitalization for investigating other probable causes with negative results. No signs of pericardial effusion recurred in a follow-up time of 12 months.

**Conclusion:**

This case report demonstrated, for the first time, that very late post-procedural cardiac tamponade might occur after catheter ablation of ventricular arrhythmias, even without antithrombotic treatment.

## Background

Cardiac tamponade is a potentially fatal complication after catheter ablation of ventricular arrhythmias (VAs). The incidence rate varies depending on the underlying heart disease of patients and procedure setting [1, 2]. Overall, cardiac tamponade is rare after catheter ablation of idiopathic premature ventricular complexes (PVCs). It often happens during or shortly after the procedure and needs urgent treatment. Here, we present a very incredible case about cardiac tamponade after catheter ablation of PVCs.

## Case report

**Fig. 1:**
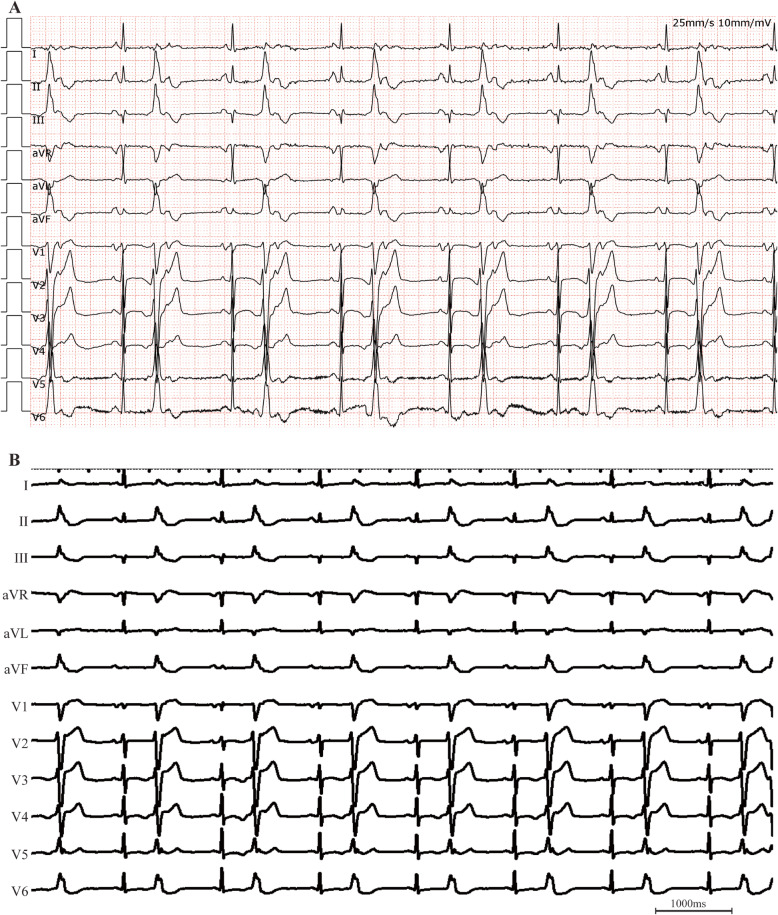
Surface ECG QRS morphology of PVCs before (**a**) and during (**b**) ablation

**Fig. 2:**
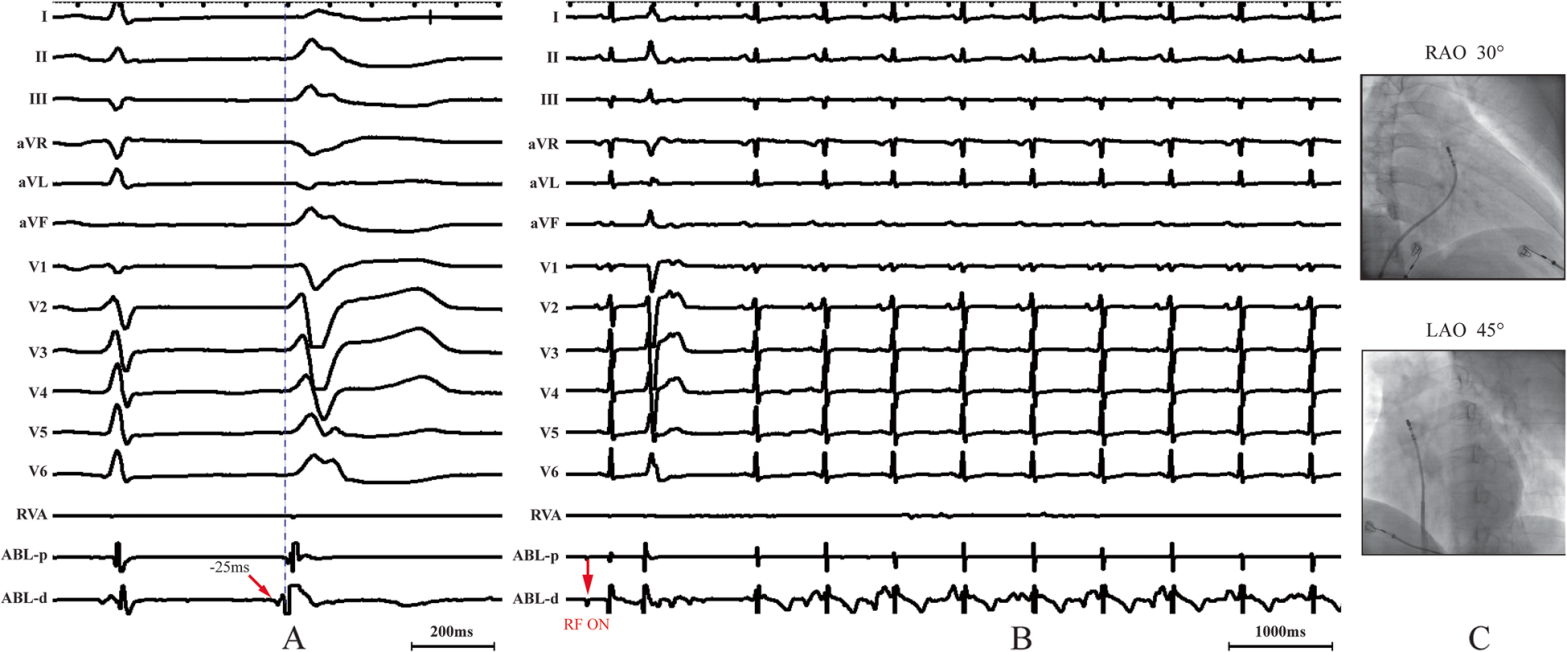
**a** Surface ECG leads along with bipolar electrograms from site of origin are displayed; **b** PVCs were terminated 1 s after the application of radiofrequency current; **c** Fluoroscopic view of catheter positioned in the RVOT

A 66-year-old woman without structural heart disease, but with a significant PVC burden of 47% (49,939/105,871 beats), was referred to the Electrophysiology Laboratory for possible catheter ablation of the PVC focus following lack of symptomatic improvement with medical treatment. The electrocardiogram (ECG) morphology of the PVCs suggested a right ventricular outflow tract (RVOT) origin (Fig. 1). A single conventional catheter guided by fluoroscopy was selected for mapping and ablation. Namely, a roving standard ablation catheter (7 French, 4-mm tip) introduced from the right femoral vein was used for location of the earliest activation site. The earliest activation timing of PVCs was identified on the posterior-lateral wall of the RVOT with a local activation time of 25 ms (Fig. 2a, c). Radiofrequency (RF) current was applied at this location. After termination of PVCs within 1 s, RF delivery continued for up to 90 s at a power setting of 30–40 W with a target temperature of 55 °C (Fig. 2b). PVCs were no longer observed for a period of 30 min during infusion of isoproterenol (4 μg/min). A total of 3000 U heparin was given during the procedure. Pericardial effusion (PE) was not evident in the post-procedure transthoracic echocardiography (TTE). She was discharged symptom-free from the hospital 1 day after the procedure (blood pressure, 124/64 mmHg; heart rate, 83 bpm), without anticoagulant or antiplatelet treatment.

**Fig. 3:**
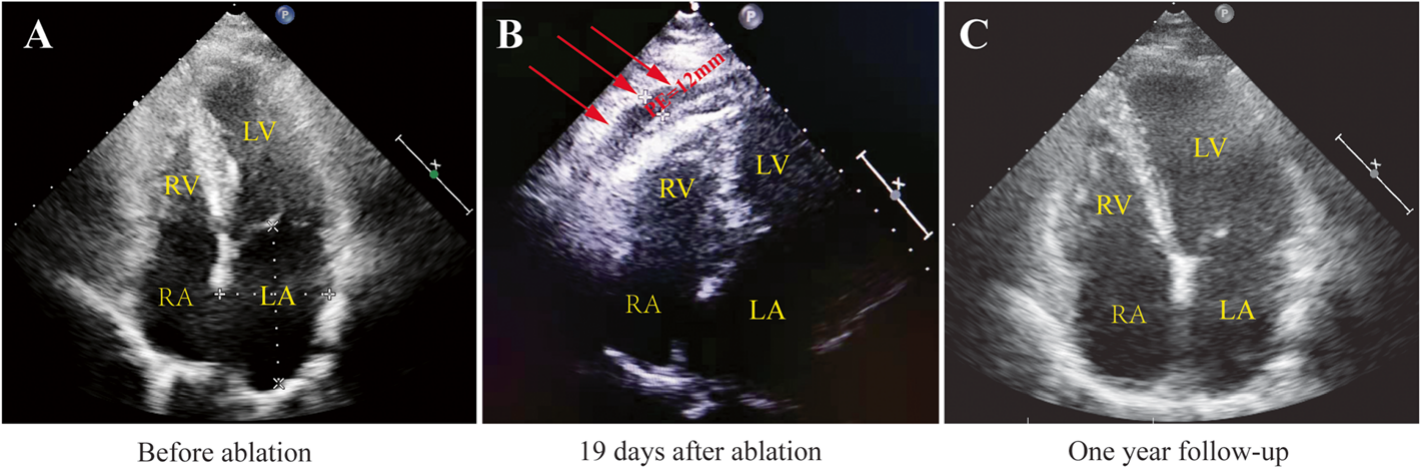
Echocardiographic images in 4-chamber view (**a**) before ablation, (**b**) at the time of cardiac tamponade, and (**c**) at 1-year follow-up are displayed

Nineteen days later, the patient was sent to our hospital after experiencing sudden syncope. She appeared pale with blood pressure 50/40 mmHg, heart rate 110 bpm, and oxygen saturation of 100% on oxygen (on 3 L oxygen per nasal cannula). Upon physical examination, her neck veins were distended, lungs were clear, and heart sounds were distant. Brain CT scans excluded cerebral hemorrhage. All blood test parameters were normal, except for an extremely elevated D-dimer (2450 μmol/L). The patient’s recent history of cardiac ablation alerted us of the possibility of cardiac problems. TTE revealed a moderate PE (12 mm) with evidence of tamponade, prompting a diagnosis of delayed cardiac tamponade (DCT) (Fig. 3b). Following an emergent pericardiocentesis to drain a hemorrhagic effusion (200 mL), the patient’s hemodynamics improved significantly. She was monitored in the Cardiac Care Unit overnight without signs of fluid re-accumulation. The next day, her condition improved, becoming conscious. On the third day of hospital admission, TTE showed no evidence of PE. She was discharged after a 2-week hospitalization for investigating other probable causes, such as cancer, infection, and autoimmune disease, with negative results. No signs of PE recurred during a 12-month follow-up (Fig. 3c).

## Discussion

DCT due to catheter ablation has been described as hypotension or cardiogenic shock requiring pericardial drainage at least 1 h after the procedure [3]. It is exceedingly rare, with the most series reports after ablation of atrial fibrillation (AF). In a global report of 27,921 ablation procedures of AF, DCT was found in 45 cases among 21,478 patients (0.2%), with a median presentation of 12 days (range 0.2–45 days) after the procedure [3]. To date, no dedicated DCT case after ablation of VAs has been reported, although there are, nonetheless, cases of cardiac tamponade during or shortly after ablation of VAs. This case report demonstrates, for the first time, that very late post-procedural cardiac tamponade can happen after catheter ablation of VAs.

The potential mechanisms of DCT include rupture of local ulcers induced by ablation or small pericardial hemorrhages due to intense post-procedural anticoagulation. Delayed rupture of the local ulcer needs relatively thin musculature to occur. One reason why DCT is most commonly encountered after AF ablation is the thin left atrial wall. Similarly, the main body of the RVOT, except the base, is formed by thin right ventricle musculature (3–5 mm thick), which makes the RVOT more likely to perforate compared to other ventricular locations, either acute or delayed. In addition, many patients with RVOT arrhythmias, previously identified as having normal cardiac structure by TTE, may in fact have latent early-stage cardiomyopathy in minute regions [4], which is also a potential risk factor for perforation. Cardiac tamponade secondary to warfarin or new oral anticoagulants has been sporadically reported [5–8]. According to case reports, it seems that spontaneous pericardial hemorrhages were prone to occur in patients with high-risk factors for bleeding, including advanced age, chronic kidney disease, and malignancy. In this case, abrupt symptom development and hemorrhagic PE are consistent with an acute process; however, the precise mechanism is unknown. We believe that the direct cause of DCT might be a tear in the myocardium, and that tear probably results from local ulcers induced by the previous ablation.

Treatment options for cardiac tamponade include pericardiocentesis and surgical repair, although conservative management could be used for small hemodynamically stable effusions. In DCT after AF catheter ablation, Cappato et al. reported that 36 cases (80%) were treated with pericardiocentesis, whereas 8 cases (17.8%) required open surgical repair [3], with the ratio similar to that reported in the multicenter study about cardiac tamponade during AF catheter ablation [9]. As DCT after ablation of VAs is rare, there is no prognostic data for reference. Therefore, large-scale retrospective studies should be conducted in the future to investigate the incidence of DCT after ablation of VAs in the real world.

Patients with DCT don’t have specific early symptoms between the procedure and cardiac tamponade, and a common sign is hypotension, which develops in minutes to days [3]. Physicians, mainly cardiologists, should always be aware of the possibility of DCT, especially in patients with sustained hypotension who have a recent history of cardiac catheter ablation.

## Conclusions

Cardiac tamponade is a potentially fatal complication after catheter ablation of VAs. It could happen very late after ablation of VAs, even without antithrombotic treatment. Large-scale retrospective studies should be conducted in the future to investigate the incidence of DCT after ablation of VAs in the real world.

## Data Availability

All relevant data supporting the conclusions of this article are included within the article.

## Abbreviations

VAs: Ventricular arrhythmias
PVCs: Premature ventricular complexes
ECG: Electrocardiogram
RVOT: Right ventricular outflow tract
PE: Pericardial effusion
TTE: Transthoracic echocardiography
RF: Radiofrequency
DCT: Delayed cardiac tamponade
AF: Atrial fibrillation
